# The role of co-infections on cervical intraepithelial neoplasia prevalence in western Kenya

**DOI:** 10.21203/rs.3.rs-4939944/v1

**Published:** 2024-10-15

**Authors:** Calleb George Onyango, Lilian Ogonda, Bernard Guyah

**Affiliations:** Maseno University; Maseno University; Maseno University

**Keywords:** Co-infections, Chlamydia trachomatis, HPV, HSV-2, HIV, CIN, Kenya

## Abstract

**Background:**

Screening for co-infections with HIV, HSV-2 and *Chlamydia trachomatis (CT)* among high-risk human papilloma virus (hr-HPV) positive women remains essential in alleviating high morbidity of cervical cancer (CC). The aim of this study was to determine the prevalence of cervical intraepithelial neoplasia (CIN) among women referred for CC screening at a referral hospital in Kisumu County, Kenya; and to establish the role of co-infection on CIN.

**Method:**

In a cross-sectional study, we collected HPV, HIV, HSV-2 and CT data, cervical cytology results, and demographic information from 517 referrals. Blood samples were obtained for HIV and HSV-2 tests; urine for CT test and cervical swabs for hr-HPV test.

**Results:**

The overall prevalence of CIN was 18.4% (95/517) with CIN1 observed in 56(29.6%), CIN2 in 27(`14.3%), CIN3 + in 12(6.3%) and normal biopsy in 94(49.7%) of the patients out of which high grade CIN2 and above (CIN2+) was 7.54% (39/517) equivalent to 32.5 per 100,000 women per year. HPV/HIV co-infection (infected vs. uninfected: OR 2.79; 95% CI 1.56–5.10, p < 0.001); HPV/HSV-2 co-infection (infected vs. uninfected: OR 2.41, 95% CI: 1.12–5.46, p < 0.024); HPV/CT co-infection (infected vs. uninfected: OR 3.83; 95% CI 1.84–8.51, p < 0.001) were found to be significantly associated with CIN.

**Conclusion:**

Overall prevalence of CIN was high in the region although high-grade CIN2 + remained relatively lower as reported earlier. Age factor, widowhood and co-infections with HIV, HSV-2 or *Chlamydia trachomatis* were associated with increased risk of testing positive for CIN.

## Introduction

Cervical intraepithelial neoplasia (CIN), the precursor of cervical cancer remain the greatest threat to the reproductive health of many women especially those living in low income, high HIV burden countries in sub Saharan Africa[1]. Globally, the disease is ranked 4th in both incidence and cancer-related mortality amongst women with an estimated 660,000 new cases and 350,000 deaths annually, and accounts for about 13.1% of all new female cancers globally [1]. However, Kenya among other East African countries remains the most affected with an estimated age-standardized incidence and mortality rates of 40.1 and per 100,000 respectively [2]

Despite improved preventive strategies including public mobilization for early screening, reduced exposure to infectious agents through community awareness campaign, enhanced immunization to targeted age group, and a concerted campaign to reduce risky behaviors that promotes the spread of the disease [3], Kenya has continued to experience higher incidence and mortality of 5236 and 3211 respectively, annually [1] with prevalence in Kisumu estimated at 8.2% [4]. Besides, infection with HIV confers susceptibility to human papilloma virus (HPV), the causative agent of cervical cancer; and women infected with HIV are at high risk of contracting the disease [5]. Women infected with HIV experience a decline in both the number and function of CD4^+^ T cells leading to high rate of contracting HPV and reduction of chance for spontaneous clearance [6]. Although Kisumu continue to register high burden of HIV [7], the prevalence of CIN in the region remain scanty.

More importantly, high risk human papilloma virus (hr-HPV), particularly HPV types 16 and 18, remain the major causative agent of most malignant and premalignant lesions of the cervix, presenting nearly 99% cases of cervical cancer [3]. The virus penetrates the basal layers of epithelial cells through micro-abrasion of the transformation zone of the cervix using two oncogenes E6 and E7 [8], but which also interact with and inhibit various cell cycle-regulating protein such as retinoblastoma gene product pRB, and p53 protein leading to cervical dyskaryosis following a series of proteolytic degradations. In Kenya, about 9.1% women in the general population are estimated to harbor cervical HPV types 16 /18 infection that also contribute to more than 63% of all invasive cervical cancers in the Country [1]. Besides, higher prevalence of 9.9% have been reported among HIV-infected women in Kisumu [9] suggesting the likelihood of higher burden of CIN in the region.

Moreover, co-infections with herpes simplex virus type 2 (HSV-2) serve as an independent predictor for cervical cancer owing to its role in facilitating HIV acquisition and transmission among sexually active community [10]. Precisely, the virus evolved strategies that counteract caspase activation and apoptosis by encoding anti-apoptotic viral proteins such as ribonucleotide reductase large subunit (R1) [11] leading to the persistence of HPV within the cervix. Although high prevalence of HIV / HSV-2 co-infection have been reported among the local fishing community [12], the role of HSV-2 / HPV co-infection in CIN remain scanty. Equally, *Chlamydia trachomatis (CT)* like other intracellular pathogens have been shown to possess the potential of altering gene expression and protein production in cervical basal cells leading to induction of host genome duplicate that results in aneuploidy and chromosome instability [13]. As *Chlamydia trachomatis* induced DNA double-strand breaks, it simultaneously inhibits proper DNA damage response and repair mechanisms rendering host cells prone to loss of genetic integrity and transformation [14]. Although higher prevalence of HIV / *Chlamydia trachomatis* co-infection have been reported in the region [15] the role of co-infections on CIN is scanty.

Elsewhere, studies have shown that hormonal contraceptives use increase the risks of developing cervical cancer especially when used for longer period of time [16]. Precisely, the upstream regulatory region (URR) of HPV 16 genome that mediate transcriptional control of HPV genes contains promoter elements that are activated by persistent interaction with steroid hormones which binds to specific glucocorticoid-response receptors within the HPV and enhance the expression of E6 and E7; which in turn bind to and degrade p53 gene product, leading to a apoptotic failure and the development of CIN [17]. Locally, adolescent girls and young women form the majority of contraceptives users with higher preference being injectable and implants [18]. We examined the influence of hormonal contraceptives on CIN prevalence in the region.

## Method

### Study design:

**Figure 1:**
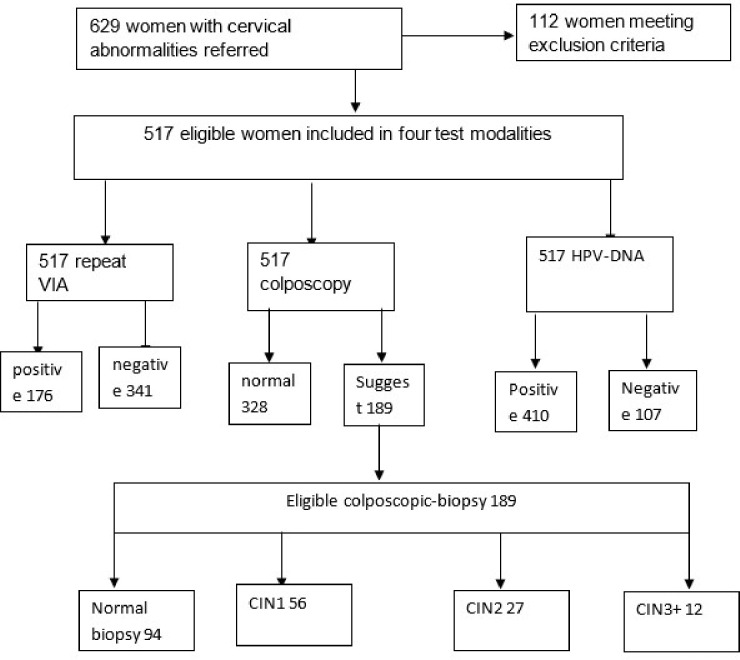
Flowchart of participants in the study

In a cross-sectional study, 517 women aged 25 to 65 years referred to Jaramogi Oginga Odinga Teaching and Referral Hospital (JOOTRH) from peripheral facilities with vaginal or cervix abnormalities between the years 2021 to 2023 were consecutively enrolled in the study after excluding 112 due to insufficient cytological samples (Fig. 1). Eligibility included: (1) women with a history of sexual activity, (2) initial visual inspection with acetic acid positive result from referral facilities and (3) informed consent. The exclusion criteria included women: (1) who were pregnant; (2) who had vaginal medication 2 days prior to the screening day (3) those with hysterectomy, muco-purulent discharge, active virginal bleeding. Nurses in the cervical screening clinic identified potential participants who attended the clinic and explained to them details of the study. Written informed consent was obtained in Kiswahili or English before study enrollment. Clinical examination involved a gynecological examination with inspection of the cervix uteri and collection of specimens by a gynecologist in a separate room. Cervical sample were collected for HPV DNA testing in PreservCyt^®^ Solution (Hologic) using the Cervex-Brush^®^ (Rover). The sample was then stored at ambient temperature until tested. Finally, the cervix was examined after the application of 5% acetic acid (VIA) and the findings recorded in datasheet. All women were informed about the VIA result immediately, but for laboratory results, they were booked to collect after one month in the next clinic review visit.

### Laboratory testing:

HIV infection was tested using Determine HIV-1/2 and confirmed with First Response HIV1/2 card tests; HSV-2 was tested using HerpeSelect-2 enzyme immunoassay; HPV tested using Gene Xpert HPV assay (Cepheid, Sunnyvale, California, United States [US]). Gene Xpert HPV is based on a multiplex real-time PCR targeting E6 and E7 oncogenes of 14 HR-HPV genotypes. The amplification was performed in five fluorescent channels that identifies five groups namely; HPV16, HPV18/45, HPV31/33/35/52/58, HPV51/59, and HPV39/56/66/68, and the results interpreted using GeneXpert software version 4.8 (Cepheid). *Chlamydia trachomatis* was tested using Gene Xpert CT/NG assay (Cepheid, Sunnyvale, California, United States [US]) with primers and probes for the detection of specific chromosomal sequence (serovariants D-K) expressed by the ompA gene of the bacteria in urine sample; while biopsy was tested using hematoxylin and eosin stains.

### Data collection

As enrollment continues, information was collected from participants on socio-demographic status and relevant sexual and reproductive health issues including HIV and ART status, contraceptive use, types and duration and the number of children. Study information was collected electronically except consent forms, a copy of which was issued to the participants after signing. The consent forms with participants’ signatures and national ID numbers were collected and secured in locked file cabinets. Labels of laboratory sample were handwritten and contained a computer-generated subject identifier and sample date.

### Statistical analysis

Continuous variables were summarized using mean and standard deviation. Categorical variables were summarized using percentages. Prevalence of CIN, HPV, HIV and HSV-2 positivity were calculated overall. A chi-square test was used to compare proportions within-group and logistic regression to estimate associations between demographic characteristics and co-infections and the prevalence of CIN.

### Ethics considerations

The study approval was obtained from the local review board at Maseno University and JOOTRH Kisumu, Kenya.

## Results

**Figure 2:**
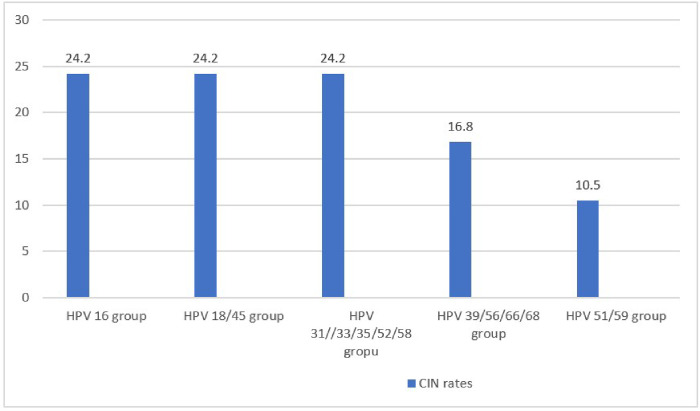
Distribution rate of CIN among hr-HPV subgroups (n = 95)

Of the 517 eligible participants, the mean age standard deviation was 34.65 ± 7.45, age range 22–57 years with a median (IQR) of 33 (28, 39) of whom majority 456(88%) were married with children ranging 3 to 4, and implant (34.4%) and injectable (39.3%) were the most preferred contraceptives with a duration median (IQR) of 11 (7, 5) years (Table 1). Among the participants examined, 123(24%), 63(12%), 48(8.9%) and 410(79%), tested positive for HIV, CT, HSV-2 and hr-HPV infections respectively. All colposcopy suggestive of CIN were further subjected to colposcopically guided biopsy for histology examination. Altogether, 189 biopsy specimens were processed and examined, out of which CIN1 were 56(29.6%); CIN2 were 27(14.3%); CIN3 + were 12(6.3%); while normal cervix were 94(49.7%). Overall, the prevalence of CIN was 18.4% (95/517) of which high grade CIN2 and above (CIN2+) was 7.54% (39/517) equivalent to 32.5 per 100,000 women per year. Of the 95 women positive for CIN lesions, hr-HPV subgoup16 and subgroup 18/45 were the most common (Fig. 2)

### Correlates associated with CIN positivity

In a Univariate analysis, the study found women aged 30 years and below (OR 0.55; 95% CI 0.22–1.31, p < 0.001) were 0.5 times less likely to test positive for CIN as compared to women above. 30 years. Besides, widows (OR 11.4; 95% CI 3.18–73.2, p < 0.001) were 11.4 times more likely to test positive for CIN, while single women [OR 0.80; 95% CI 0.10–4.96, p = 0.300) were 0.80 times less likely to test positive for CIN as compared to married women. Women co-infected with HIV (OR = 2.79; 95% CI 1.56–5.10, p < 0.001); were 2.8 times more likely to test positive for CIN as compared to HIV uninfected counter parts. Additionally, women co-infected with HSV-2 (OR = 2.41, 95% CI: 1.12–5.46, p < 0.024); were 2.4 times more likely to test positive for CIN as compared to HSV-2 uninfected counter parts. Women co-infected with *Chlamydia trachomatis* (OR = 3.83; 95% CI 1.84–8.51, p < 0.001); were 3.8 times more likely to test positive for CIN as compared to uninfected counter parts (Table 2)

## DISCUSSION

The present study was set to determine the prevalence and determinants of CIN among women in Kisumu County, Kenya. Although continuous monitoring and reporting on prevalence of CIN is essential for estimating the risk of developing cervical cancer and optimizing screening strategy for early detections and treatment [3] this is the first study to comprehensively examine the carriage rate of CIN exclusively in HIV endemic region of western Kenya.

Findings of the study revealed that the overall prevalence of cervical intraepithelial neoplasia (CIN) was 18.4% (95/517) of which high grade CIN2 and above (CIN2+) was 7.54% (39/517) equitable to approximately 32.5 per 100,000 women per year; relatively lower than the national incidence of 40.1 per 100, 000 women per year [2],as well as neighbourhood Uganda (56.2 per 100,000 women per year) [19] and Tanzania (54.0 per 100, 000 women per year) [20]. Although previous estimate had reported higher prevalence of 27.9% severe dysplasia in the same region [21] as well as 21.4% prevalence in Nairobi County [22], our finding was within the range of 3.7% – 22.6% reported around Africa [23] and comparable to 8.2% prevalence report earlier by Mungo et al in the same region [4] suggesting the disease could be widespread locally. This finding is a reflection of high HPV prevalence of 9.1% in the general population [1] as well as 51.1% prevalence among sexually active men of Kisumu County [24] majorly attributed to HPV subtypes 16 and 18 [25]. In this study, hr-HPV subgroup 16 and 18/45 were the most prevalent and major cause of CIN in the region contributing to nearly half of all new cases detected. Higher prevalence of HPV genotype 16 have been reported earlier in the same region [5] as well as in Nairobi [22] thus affirming subtype 16 as key player in CIN prevalence locally.

Analysis of risk determinants revealed that women aged 30 years and below were less likely to test positive for CIN compared to their counterpart above 30 years. However, widows were ten times more likely to test positive compared to their married counterpart. Studies have shown that being married is associated with early diagnosis of CIN and a more favorable prognosis for cervical cancer [26] possibly owing to partner support in regular clinic visit for screening and treatment. In this study, we found age factor and widowhood significantly associated with higher Odds of developing CIN. Similar observations have been recorded by Orang’o and Pinheiro [5, 26] although early onset of CIN among teenagers have also been observed in the region, possibly attributed to changes in diet, lifestyle, obesity, environment and the microbiome all of which interact with genomic and genetic susceptibilities [27]. However, parity and hormonal contraceptive use were found not associated with CIN.

Further analysis of risk factors revealed that women co-infected with HIV were twice more likely to test positive for CIN compared to their HIV-uninfected counterparts suggesting that, co-infection with HIV is independently and significantly associated with higher Odds of developing CIN. Similar observation have been reported by Perez-Gonzalez *et al* and Sosso et al all noted that HPV infection was common among people living with HIV (PLWH) who were at greater risk of developing CIN [6, 28]. Indeed, PLWH have been shown to experience increased risk of persistent HPV infection including high viral load essential for CIN aetiology [29] more so when co-infection involve multiple HPV genotypes [22]. Few studies have explored the relationship between HSV-2 co-infection and the severity of cervical lesion. In our study, women co-infected with HSV-2 were twice more likely to test positive for CIN compared to their HSV-2-uninfected counterparts suggesting that co-infection with HSV-2 is significantly associated with higher Odds of developing CIN. It’s good to note that HSV-2 co-infection has been incriminated with the initiation of oncogenic processes that are eventually picked by HPV in driving cervical cancer development [30]. Our finding corroborated a systematic review and a meta-analysis by Zhang *et al* [31] showing that women co-infected with HSV-2 were 3 times more likely to test positive for CIN. Elsewhere, in another systematic review, higher frequency of HSV-2 was observed among women experiencing invasive cervical cancer [30] suggesting a possible association that could potentially be attributed to shared immunological compromise, genetic predisposition, or lifestyle factors. In other studies, it has been suggested that genital HSV-2 infection possibly act in conjunction with HPV infection to increase modestly the risk of cervical intraepithelial neoplasia [32] of which investigation is still in progress.

Women co-infected with *Chlamydia trachomatis (CT)* were three times more likely to test positive for CIN compared to their CT-uninfected counterparts suggesting that co-infection with CT is equally associated with higher Odds of developing CIN. Again, this was in concurrence with a finding by Lu *et al*., showing prevalence of 21.8% low grade squamous intraepithelial lesions (LSIL) and 10.8% high grade squamous intraepithelial lesions (HSIL) among women co-infected with CT / HPV [33], and consistent with another study from neighborhood Uganda recording a significant association between CT / HPV co-infection and the development of LSIL [23]. More importantly, studies have shown that co-infection with CT supports HPV persistence by suppressing the functions of Langerhans cells (LCs) pathways which are involved in the regulation of immune responses. Besides, the infection impairs LC functions by reducing the antigen-presenting ability and density of LCs; alter T-cell subsets, resulting in fewer CD4 + and CD8 + T cells and more infiltrating Tregs; decreases the CD4+/CD8 + T cell ratio to below 1; and induces greater T lymphocytes’ apoptosis, hence impairing cell-mediated immunity and accelerating the progression of CIN [33]. In Rome Italy, women co-infected with *CT / HPV* were found to experience high frequency of high grade cervical lesions compared to their counterparts infected with HPV only [34] suggesting the important role played by CT in cervical carcinogenesis.

It is also good to note that previous local studies have mainly focused on the relationship between cervical cytology results and sexually transmitted pathogens majorly HIV and HPV [5, 22]. Conversely, cervical cytology results do not adequately represent the true picture of the cervix. Instead, cervical biopsy provides the gold standard for assessing cervical abnormalities, highlighting the need for assessing the relationship between sexually transmitted pathogens, HPV infection, and histological findings. In our study, we found a strong association between HIV, *CT* and HSV-2 co-infection with hr-HPV and prevalence of CIN. This study had some limitations. First, the participants were recruited from a gynecological clinic having been referred from peripheral facilities with either vaginal or cervical abnormalities. This facility-based recruitment coupled with structural inefficiency in some rural setting that conveyed referrals limit the generalization of the study findings.

### Conclusion

The overall prevalence of CIN was high in the region although high-grade CIN2 and above (CIN2+) remained relatively lower as reported earlier. Age factor, widowhood and co-infections with HIV, HSV-2 or *Chlamydia trachomatis* were associated with increased risk of testing positive for CIN, but not parity or hormonal contraceptives use.

## Figures and Tables

**Table 1:** Demographic and clinical characteristics of study participants

		Histological diagnosis				
Variable	Overall, N = 517^1^	CIN1, N = 56^1^	CIN2, N = 27^1^	CIN3+, N = 12^1^	normal, N = 94^1^	unavailable, N = 328^1^
**Age**	33 (28, 39)	38(34,46)	46(39,48)	43(39,4)	37(32,42)	30 (27, 36)
**Marital status**						
Married	456 (88%)	46 (82%)	18 (67%)	10 (83%)	89 (95%)	293 (89%)
Single	35 (6.8%)	1 (1.8%)	0 (0%)	1(8.3%)	3 (3.2%)	30 (9.1%)
Widow	26 (5.0%)	9 (16%)	9 (33%)	1(8.3%)	2 (2.1%)	5 (1.5%)
**Parity**	3.0(3.0,4.0)	4.0(3.0,4)	4.0(4.0,5)	4.0(3.0,5.0)	4.0(3.0,5)	3.0(3.0, 4.0)
**Contraceptive's type**						
Hormonal-IUD	29 (5.6%)	4 (7.1%)	3 (11%)	0 (0%)	2 (2.1%)	20 (6.1%)
Implant Jadele / Norplant	179 (35%)	24 (43%)	8 (30%)	3 (25%)	36 (38%)	108 (33%)
injectable /DepProvera	202 (39%)	18 (32%)	6 (22%)	5 (42%)	43 (46%)	130 (40%)
None	50 (9.7%)	9 (16%)	10 (37%)	3 (25%)	9 (9.6%)	19 (5.8%)
Oral pill	57 (11%)	1 (1.8%)	0 (0%)	1 (8.3%)	4 (4.3%)	51 (16%)
**Duration (Yrs.)**	11 (7, 15)	13(11,17)	13 (0, 17)	15 (8,18)	13(10,19)	10 (7, 13)
**VIA**						
negative	341 (66%)	35 (63%)	11(41%)	5 (42%)	58 (62%)	232 (71%)
positive	176 (34%)	21 (37%)	16(59%)	7 (58%)	36 (38%)	96 (29%)
**HPV test**						
negative	107 (21%)	3 (5.4%)	2 (7.4%)	1 (8.3%)	12(12.8)	89 (27%)
positive	410 (79%)	53(94.6)	25(92.6)	11(91.7%)	82 (87%)	239 (73%)
**HIV status**						
negative	394 (76%)	22 (39%)	12(44%)	6 (50%)	63 (67%)	291 (89%)
positive	123 (24%)	34 (61%)	15(56%)	6 (50%)	31 (33%)	37 (11%)
**HSV-2**						
negative	471(91%)	40(71%)	23(85%)	9(75%)	83(88%)	316(96%)
positive	46(8.9%)	16(29%)	4(15%)	3(25%)	11(12%)	12(3.7%)
**Chlamydia**						
Negative	454 (88%)	35 (63%)	20(74%)	8 (67%)	83 (88%)	308 (94%)
Positive	63 (12%)	21 (38%)	7 (26%)	4 (33%)	11 (12%)	20 (6.1%)

CIN1, CIN2, CIN3: Cervical intraepithelial neoplasia 1, 2 or3, HSV2: herpes simplex virus 2,

1 Median (IQR); n (%).

**Table 2:** Correlates associated with CIN positivity by demographic and clinical characteristics

	Univariate					Multivariable		
Characteristic	N	Event N	OR^1^	95% CI^1^	p-value	OR^1^	95% CI^1^	p-value
**Age category**	189	95			0.18			
> 30 years			*ref.*	–		*ref.*	–	
≤ 30 years			0.55	0.22–1.31		0.20	0.08–0.47	**< 0.001**
Marital status	189	95			**< 0.001**			
Married			*ref.*	–		*ref.*	–	
Single			0.80	0.10–4.96		2.55	0.35–12.3	0.303
Widow			11.4	3.18–73.2		12.3	3.70–46.5	**< 0.001**
**parity**	189	95			0.460			
> 4 children			*ref.*	–				
≤ 4 children			0.79	0.42–1.47				
**Contraceptives types**	189	95			**0.017**			
Hormonal-IUD			*ref.*	–		*ref.*	–	
Implant Jadele / Norplant			0.28	0.04–1.24		1.79	0.57–6.43	0.302
injectable / Depoprovera			0.19	0.03–0.86		1.24	0.39–4.42	0.700
None			0.70	0.09–3.60		1.35	0.33–5.85	0.711
Oral pill			0.14	0.01–1.27		0.80	0.10–4.92	0.801
**Contraceptive duration**	189	95			0.700			
> 20			*ref.*	–				
≤ 20 years			0.86	0.38–1.92				
**HIV status**	189	95			**< 0.001**			
negative			*ref.*	–		*ref.*	–	
positive			2.79	1.56–5.10		4.45	2.53–7.92	**< 0.001**
**HSV2**	189	95			**0.024**			
Negative			*ref.*	–		*ref.*	–	
Positive			2.41	1.12–5.46		5.67	2.61–12.4	**< 0.001**
**Chlamydia**	189	95			**< 0.001**			
Negative			*ref.*	–		*ref.*	–	
Positive			3.83	1.84–8.51		6.03	3.00–12.2	**< 0.001**

Regression analysis.

1 OR = Odds Ratio; CI = Confidence Interval; ref = reference; HSV-2, Herpes simplex virus-2; HPV, Human papilloma virus.

## Data Availability

The datasets used or analyzed during the current study are available from the corresponding author on reasonable request

## Abbreviations

CI: confidence interval
SD: standard deviation
VL: viral load
CT: *Chlamydia Trachomatis*
CC: Cervical Cancer
CIN 1, 2, 3: Cervical Intraepithelial Neoplasia (Grade 1, Grade 2, Grade 3)
ICO-IARC: Catalan Institute of Oncology / International Agency for Research on Cancer
HPV: Human Papilloma Virus
HIV: Human Immunodeficiency virus
HR-HPV: High Risk Human Papilloma Virus
JOOTRH: Jaramogi Oginga Odinga Teaching and Referral Hospital
